# Genome Wide Analysis of Chromosomal Alterations in Oral Squamous Cell Carcinomas Revealed over Expression of MGAM and ADAM9

**DOI:** 10.1371/journal.pone.0054705

**Published:** 2013-02-06

**Authors:** Vui King Vincent-Chong, Arif Anwar, Lee Peng Karen-Ng, Sok Ching Cheong, Yi-Hsin Yang, Padmaja Jayaprasad Pradeep, Zainal Ariff Abdul Rahman, Siti Mazlipah Ismail, Zuraiza Mohamad Zaini, Narayanan Prepageran, Thomas George Kallarakkal, Anand Ramanathan, Nur Aaina Binti Mohd Mohayadi, Nurul Shielawati Binti Mohamed Rosli, Wan Mahadzir Wan Mustafa, Mannil Thomas Abraham, Keng Kiong Tay, Rosnah Binti Zain

**Affiliations:** 1 Oral Cancer Research and Coordinating Centre, Faculty of Dentistry, University of Malaya, Kuala Lumpur, Malaysia; 2 Department of Oral and Maxillofacial Surgery, Faculty of Dentistry, University of Malaya, Kuala Lumpur, Malaysia; 3 Sengenics Sdn Bhd, Petaling Jaya, Selangor Darul Ehsan, Malaysia; 4 Oral Cancer Research Team, Cancer Research Initiatives Foundation, Selangor Darul Ehsan, Malaysia; 5 Department of Dental Hygiene, College of Dental Medicine, Kaohsiung Medical University, Kaohsiung, Taiwan; 6 Department of Oral Pathology, Oral Medicine and Periodontology, Faculty of Dentistry, University of Malaya, Kuala Lumpur, Malaysia; 7 Department of Otorhinolaringology, Faculty of Medicine, University of Malaya, Kuala Lumpur, Malaysia; 8 Oral Health Division, Ministry of Health, Putrajaya, Malaysia; Beijing Tiantan Hospital, Capital Medical University, China

## Abstract

Despite the advances in diagnosis and treatment of oral squamous cell carcinoma (OSCC), mortality and morbidity rates have not improved over the past decade. A major drawback in diagnosis and treatment of OSCC is the lack of knowledge relating to how genetic instability in oral cancer genomes affects oral carcinogenesis. Hence, the key aim of this study was to identify copy number alterations (CNAs) that may be cancer associated in OSCC using high-resolution array comparative genomic hybridization (aCGH). To our knowledge this is the first study to use ultra-high density aCGH microarrays to profile a large number of OSCC genomes (n = 46). The most frequently amplified CNAs were located on chromosome 11q11(52%), 2p22.3(52%), 1q21.3–q22(54%), 6p21.32(59%), 20p13(61%), 7q34(52% and 72%),8p11.23–p11.22(80%), 8q11.1–q24.4(54%), 9q13–q34.3(54%), 11q23.3–q25(57%); 14q21.3–q31.1(54%); 14q31.3–q32.33(57%), 20p13–p12.3(54%) and 20q11.21–q13.33(52%). The most frequently deleted chromosome region was located on 3q26.1 (54%). In order to verify the CNAs from aCGH using quantitative polymerase chain reaction (qPCR), the three top most amplified regions and their associated genes, namely ADAM5P (8p11.23–p11.22), MGAM (7q34) and SIRPB1 (20p13.1), were selected in this study. The ADAM5P locus was found to be amplified in 39 samples and deleted in one; MGAM (24 amplifications and 3 deletions); and SIRPB1 (12 amplifications, others undetermined). On the basis of putative cancer-related annotations, two genes, namely ADAM metallopeptidase domain 9 (ADAM9) and maltase-glucoamylase alpha-glucosidase (MGAM), that mapped to CNA regions were selected for further evaluation of their mRNA expression using reverse transcriptase qPCR. The over-expression of MGAM was confirmed with a 6.6 fold increase in expression at the mRNA level whereas the fold change in ADAM9 demonstrated a 1.6 fold increase. This study has identified significant regions in the OSCC genome that were amplified and resulted in consequent over-expression of the MGAM and ADAM9 genes that may be utilized as biological markers for OSCC.

## Introduction

Oral squamous cell carcinoma (OSCC) is one of the major causes of cancer-related mortality with an estimation of more than 275,000 new cases and over 120,000 deaths per year [1]. Despite numerous advances in diagnosis and treatment of oral cancer, mortality and morbidity rates for OSCC are exceedingly high. A major drawback in diagnosis and treatment of OSCC is the lack of detailed understanding of the role of genetic instability in oral carcinogenesis [2], [3].

Genomic re-organizations play an important role in the pathogenesis of cancer. A successive process of acquired genetic and epigenetic alterations from a single precursor cell is one of the hallmarks in tumour development. Changes in copy number through non-homologous recombination events result in translocations, insertions or deletions due to re-assortment of exons between different genes [4], [5] which increases the probability of acquisition of new domains for proteins, fusion transcripts resulting in potentially new or modified protein functions [6]. Other specific structural alterations can also result in activation of oncogenes or inactivation of tumour suppressor genes [7]. This has been identified in numerous types of lymphomas, leukemias and solid tumors [7]. Recently, large-scale genomics studies have identified ubiquitous prevalence of deletions and amplifications in various cancer genomes [8]. These are mostly the result of the extensive genomic re-organisation that occurs during tumorigenesis. Identifying CNAs and how they may be implicated in OSCC is the key objective of this study.

Recently, array comparative genomic hybridization (aCGH) has been utilized as a first tier diagnostic tool for genomic profiling to investigate copy number alterations of various genetic diseases such as autism, multiple congenital anomalies and developmental delay [9]. The application of aCGH for identifying DNA copy number “signatures” or profiling has been carried out in OSCC studies [3]. Although, previous studies in OSCC have used aCGH, they only used low-resolution BAC clone aCGH or low-density oligonucleotide aCGH (4×44 k and 1×105 k oligonucleotide based aCGH). As further clarification, these technologies are not able to detect micro-genomic amplications and deletions (below 30 kb) that could be missed on a targeted BAC clone [10]. In contrast, our usage of ultra-dense (1 million probe) aCGH technology has a density that is at least 10-fold higher than previous studies, enabling detailed information even down to individual exons [11]. Hence, the key aim of this study was to identify CNAs in OSCC using ultra-dense, high-resolution aCGH. To identify the specific genes within the candidate genomic regions, an independent method of copy number determination by qPCR was used in an additional independent set of OSCC tumor samples. Selected amplified genes and their mRNA expression were determined using reverse transcriptase quantitative PCR (RT-qPCR) in the CNAs regions.

## Materials and Methods

### Tumor Samples

Forty-six OSCC frozen tissues were included for the genome wide screening study using aCGH. In order to validate the CNAs from aCGH, 48 OSCC samples (12 OSCC samples overlapped with aCGH samples and an independent set of 36 OSCC samples) were used. For mRNA expression quantitation study using RT-qPCR, 30 OSCC (11 OSCC samples overlapped with aCGH samples and an independent set of 11 OSCC with 4 normal oral mucosal from non-cancer patients) were employed in this study. All fresh frozen OSCC tissues and the associated socio-demographic and clinico-pathological data including age, sex, ethnicity, and site of lesion were obtained from the Malaysian Oral Cancer Database and Tissues Bank System (MOCDTBS) coordinated by the Oral Cancer Research and Coordinating Centre, University of Malaya [12]. The site of lesion of the OSCC is classified according to the anatomical subsites of the International Classification of Disease (ICD-10), a coding system that was developed by World Health Organization (WHO) [13]. Details of the socio-demographical and clinico-pathological data of this study cohort are summarized in Table 1. Written informed consent was obtained before patients were recruited and specimens were collected, stored and later use for in this study. This study was approved by the Medical Ethics Committee (MEC), Faculty of Dentistry, University of Malaya with the MEC code no: DF0306/001/(L).

**Table 1:** Socio-demographic and clinic-pathologic parameters of 46 OSCC patients.

Sociodemographic Parameters		No. of patients n = 46 (%)
**Gender**	Male	18 (39.1%)
	Female	28 (60.9%)
**Ethnicity**	Malay	12 (26%)
	Chinese	9 (19.6%)
	Indian	21 (45.7)
	Others	4 (8.7%)
**Risk Habits**	Smoking	12 (26%)
	Drinking Alcohol	12 (26%)
	Betel Quid Chewing	22 (48%)
**Clinico-pathologic Parameters**		
**Subsites**	Tongue	20 (43.5%)
	Buccal Mucosa	17 (37%)
	Gum	7 (15.2%)
	Floor of Mouth	2 (4.3%)

### DNA and RNA Extraction

All tumor tissues were surgical excision specimens and immediately snapped frozen in liquid nitrogen. The normal tissues were obtained from the excessive flap during minor surgical of the impacted wisdom tooth. The fresh snapped frozen tumor tissues were cryo-sectioned and stained with haematoxylin and eosin (H&E) for histological assessment by oral pathologists (TG and RBZ). After histological confirmation those samples that had a tumor cell content greater than 70%, were further cryo-sectioned and were used directly for DNA and RNA extraction from the whole tissue using DNEasy Blood & Tissue Kit (Qiagen, GmBH Germany) and RNeasy micro kit (Qiagen, Hilden, Germany), respectively, according to the manufacturer’s instructions. The quality (A260/A280, A260/230) and concentration of the gDNA (ng/µl) was determined using the Nanodrop spectrophotometer ND-2000 (NanoDrop Technologies, Wilmington, DE, USA). The integrity of RNA was assessed using the Agilent Bioanalyzer**-**2100 (Agilent, Palo Alto, CA, USA) and only samples with RNA integrity number (RIN) more than 6 was included used for cDNA synthesis that was subsequently used for quantitative PCR (qPCR) analyses.

### Array CGH

Array-CGH was carried out using the SurePrint G3 Human CGH 1×1 M array (Agilent Technologies, Santa Clara, CA, USA) for genome wide survey according to the manufacturer’s protocol by Oxford Gene Technology as described previously [14]. The array slides used were oligonucleotide-based microarrays which containing 974,016 probes that enable molecular profiling of genomic imbalances with 2.1 kb average resolution. The length of each probe was 60-mer and covers both non**-**coding and coding regions of the human genome. Briefly, for each experiment, a total of 1.5µg gDNA of patient sample and 1.5µg sex matched reference blood gDNA (Promega, Madison, WI) were labelled with fluorescence Cy3 and Cy5 respectively using the CytoSure Genomic DNA labelling kit (Oxford Gene Technology, UK). The probes were purified using Microcon Centrifugation Filters, Ultracel YM-30 (Millipore, Billerica, MA, USA) and mixed together following denaturation and pre-annealing with 50µg of human Cot-1 DNA (Invitrogen, California). The mixture was then hybridized to the array slide, where hybridization was performed at a constant rotation of 20 rpm at 65°C for 40 hours. After hybridization, slides were washed with Agilent wash buffer 1 and 2 according to the manufacturer’s protocol. Slides were then scanned immediately using Agilent Microarray Scanner (Agilent Technologies, USA). The data were extracted from scanned images using Feature Extraction software, version 10.7.3.1 (Agilent Technologies, USA). Normalisation was applied using the software to reduce the inconsistencies and dye incorporation bias. The data was segmented using a modified Circular Binary Segmentation (CBS) algorithm [15]. Genomic aberrations were identified by applying a threshold log_2_ ratio value of 0.3 for gains and 0.6 for losses. This ratio was taken because a heterozygous loss of chromosomal material will result in a theoretical ratio of 2∶1, while a single amplification will give a ratio of 3∶2. In order to consider a segment as an aberration, a minimum of 10 probes were required in that segment. CNAs were reported in accordance to the human genome sequence assembly Build 36, Hg 18 (www.ncbi.nlm.nih.gov). CNAs were analysed using the population analysis feature in the Cytosure software (Oxford Gene Technology, Oxford, UK). All statistical analyses were performed using the SPSS statistical package (SPSS version 12.0, Chicago, IL) where *p* values <0.05 was considered significant.

### Copy Number Analysis by the TaqMan PCR Assay

Copy number analysis of ADAM5P, MGAM and SIRBP1 were performed for tumor DNA, which comprised of an independent set of 36 OSCC and 12 OSCC samples that overlapped with aCGH samples. The analysis was done and according to the manufacturer’s instructions and was processed in an ABI 7500 Fast Real Time PCR System (Applied Biosystems, Foster City, CA, USA). The gDNA from a healthy volunteer served as calibrator control for the analysis. Each DNA sample was analysed in quadruplicate by duplex Taqman real-time polymerase chain reaction assays. Three assays were selected for copy number analysis, which were ADAM5P (Hs03268783_cn), MGAM (Hs04340413_cn) and SIRPB1 (Hs04057639_cn). The reaction mixtures (20µl) used for amplification were 4µl of genomic DNA(5 ng/µl), 10µl of 2× TaqMan® Genotyping Master Mix (Applied Biosystems, Foster City, CA, USA), 1 µl of 20× Taqman Copy number assay, 1 µl of 20× Taqman copy number reference assay (*RNAse P*) and 4 µl of nuclease free water. PCR cycling conditions were as described in the manufacturer’s instructions. The gene copy number per diploid genome was calculated using the equation 2× (2−ΔΔCt), comparative C_T_ (ΔΔC_T_) relative quantitation method [16]. For the copy number calculation, the mean of quadruplicate were used for the target assay. For the reference assay, *RNase P*, a reference of known copy number (copy number = 2) with a calibrator sample for each target gene was taken. *RNase P* gene is known to exist only in two copies in a diploid genome. The relative quantity calculated was multiply by a base copy number of 2 to derive the copy number value. For a copy number which less than the 1 would be considered as deletion with one copy number and a copy number more than 2 as amplification [17], [18].

### mRNA Expression of ADAM9 and MGAM by RT-qPCR

Nineteen out of 46 samples used in aCGH study were combined with an independent set of 11 OSCC and 4 normal oral mucosal frozen tissues from non-cancer patients for RT-qPCR analysis. This was used to quantify mRNA expression of ADAM9 (ADAM metallopeptidase domain 9) and MGAM (maltase-glucoamylase alpha-glucosidase). Total RNA was reverse transcribed using the High Capacity cDNA reverse transcription kit (Applied Biosystems, Foster City, CA, USA). qPCR reactions were performed in triplicates on a 7500 Fast Real-Time PCR System (Applied Biosystems, Foster City, CA, USA). The TaqMan Gene Expression Assay (Applied Biosystems, Foster City, CA, USA) was performed for 2 genes; ADAM9 (Hs00177638_m1) and MGAM (Hs01090216_m1). All qPCRs were carried out according to the manufacturer’s protocol (Applied Biosystems, Foster City, CA, USA). The relative quantification/fold change (RQ) of all genes was calculated using the 2^−ΔΔCT^ method using 7500 Fast System SDS Software 1.3.1 (Applied Biosystems, Foster City, CA, USA). The housekeeping gene (GAPDH) was used as an endogenous control, while the cDNA from normal oral mucosa tissue (RQ = 1) was utilized to normalise the test samples (OSCC) and any fold change from OSCC with higher than 1 was considered over-expressed. The Mann-Whitney U test was used to compare ADAM9 and MGAM mRNA expression level in tumor and normal tissues. All statistical analyses were performed using the SPSS statistical package (SPSS version 12.0, Chicago, IL) where *p* values <0.05 was considered significant.

## Results

### Copy Number Alterations

**Figure 1:**
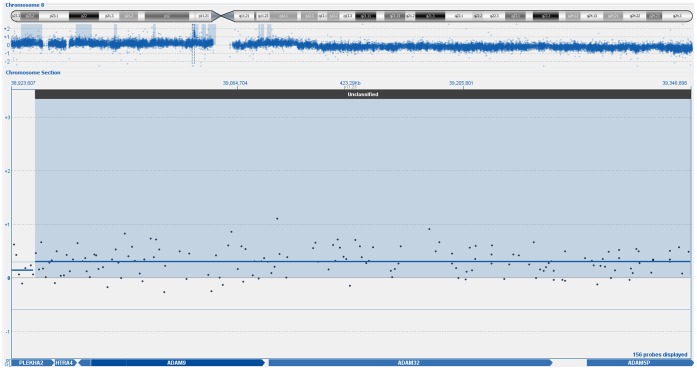
Array CGH based identification of amplified chromosome 8p11.23-11.22 region encompassing ADAM9 gene.

**Figure 2:**
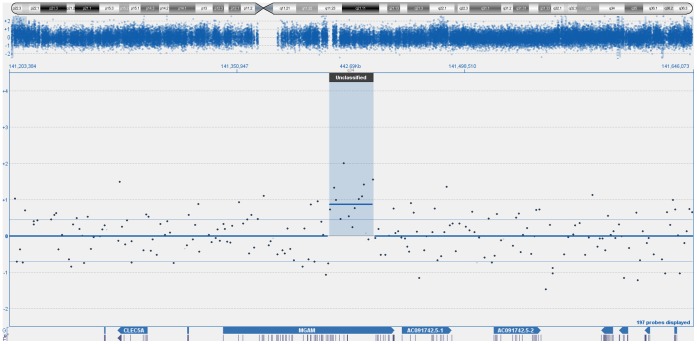
Array CGH based identification of amplified chromosome 7q34 region encompassing MGAM gene.

**Figure 3:**
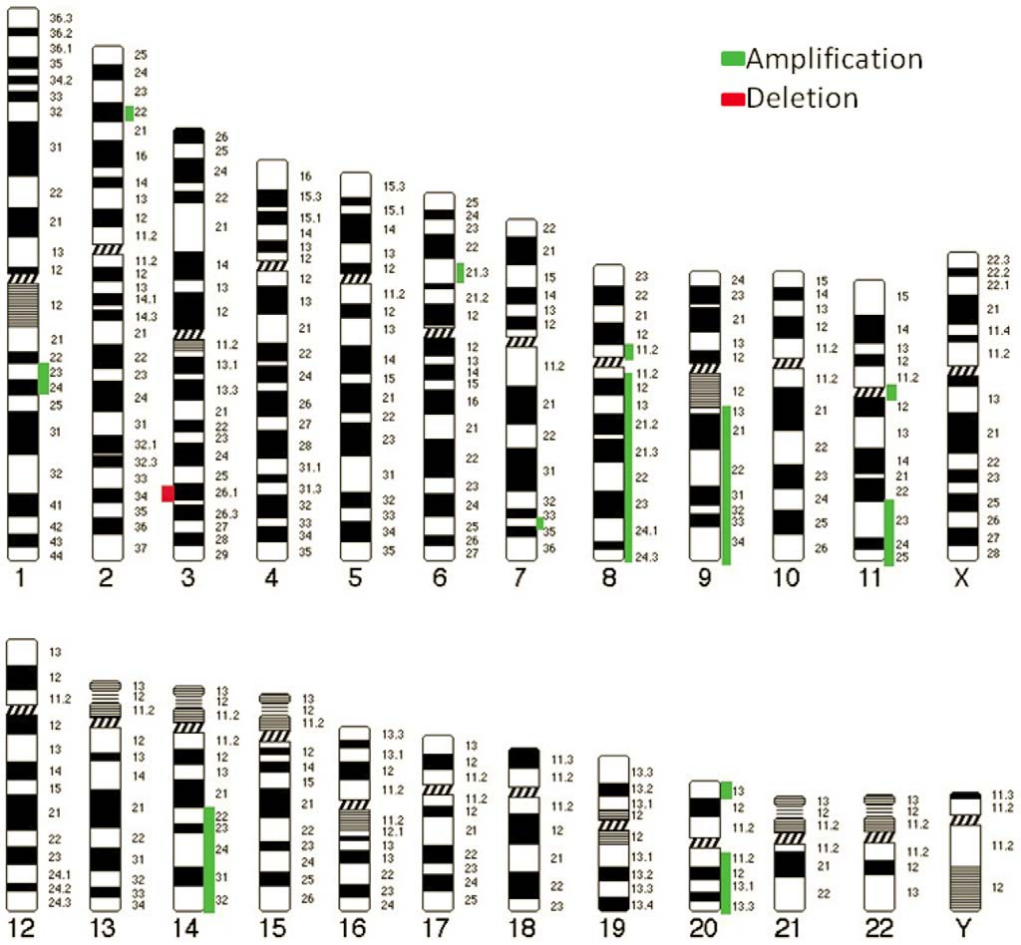
The ideogram of top most frequently CNAs in term of amplifcations and deletions identified in this study using aCGH.

Genome wide analysis using aCGH identified 47 genomic regions in the 46 OSCC cancer genome samples. Filtering was done on the basis of the log ratio and probe incidence described above. Comparison with a list of 38 previously identified and published CNAs in OSCC [19], indicated a concordance of at least 25.5%. The concordance number is ambigous as previous studies have used methods with a resolution that is at least ten-fold lower. Hence likely to under or over report CNAs and their positions. A particular feature of the results was the heterogeneity of the samples. Exactly 1/3 of the CNAs were present in a quarter of all samples. Whereas only 16 out of 47 CNAs were present in more than half of the samples (n = 46 OSCC). The top most amplified regions were on chromosome 8p11.23–p11.22 (n = 37, 80%), 7q34 (n = 24, 52%; n = 34, 74%), 20p13 (n = 28, 61%); 6p21.32 (n = 27, 59%), 1q21.3–q22 (n = 25, 54%), 11q11 (n = 24, 52%) 2p22.3 (n = 24, 52%), 8q11.1–q24.4 (n = 25, 54%), 9q13–q34.3 (n = 25, 54%), 11q23.3–q25 (n = 26, 57%); 14q21.3–q31.1 (n = 25, 54%); 14q31.3–q32.33 (n = 26, 57%), 20p13–p12.3 (n = 25, 54%) and 20q11.21–q13.33 (n = 24, 52%) whereas the deleted region was 3q26.1 (n = 25, 54%). The percentages in parentheses indicate the frequency of each event. The most frequently amplified region, 8p11.23–p11.22 contained several ADAM family genes (ADAM9, ADAM5P and ADAM32). Interestingly, almost the entire p-arm of chromosome 8 was amplified (Figure 1). The second most frequently amplified CNA, which is located on chromosome 7q34 (Figure 2) contains the MGAM gene. In contrast to the ADAM9 amplification, the MGAM genomic amplification is tightly positioned only at the gene locus. The detailed list of candidate gene(s) for all the chromosomal aberrations is shown in File S1. The top most 17 CNAs regions were also illustrated in Figure 3. All the CNAs showed no significant correlation with socio-demographical and clinic-pathological parameters.

### Taqman Copy Number Assay of ADAM5P, MGAM and SIRPB1

**Figure 4:**
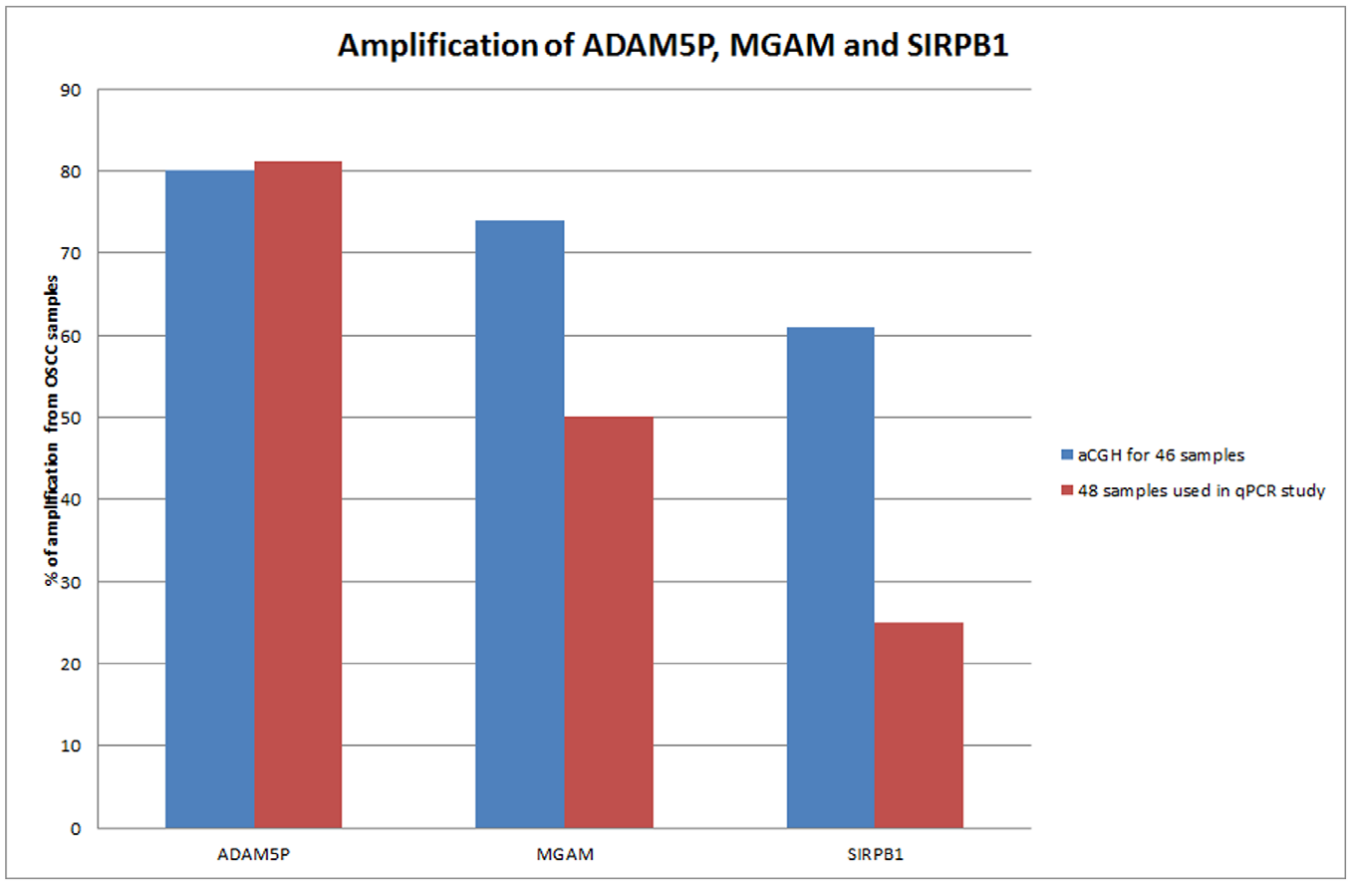
Amplification of ADAM5P, MGAM and SIRPB1 identifed by using aCGH and qPCR in OSCC samples. The percentage for amplification of ADAM5P were almost similar from aCGH and qPCR study with 80% (37/46 OSCC samples) and 81.25% (39/48 OSCC samples), respectively. Similarly, the percentage of the amplification detected from aCGH and qPCR for MGAM gene were also reported as 74% (37/46 OSCC samples) and 50% (24/48 OSCC samples), respectively. The percentage of amplification identified from aCGH and qPCR for SIRPB1 were 61% (28/46 OSCC samples) and 25% (12/48 OSCC samples).

Top three most amplified gene(s) (ADAM5P, MGAM and SIRPB1) were validated using copy number assay analysis with 48 OSCC samples which comprising of 12 OSCC which overlapped with aCGH samples and an independent sample set of 36 OSCC samples. The amplification of the selected genes identified from aCGH and qPCR were illustrated in Figure 4. The amplification of ADAM5P were almost similar between aCGH and qPCR validation with 80% (37/46 OSCC samples) and 81.25% (39/48 OSCC samples), respectively. Similarly, the amplification detected for MGAM gene were 74% (37/46 OSCC samples) in aCGH and 50% (24/48 OSCC samples) in qPCR validation. However, for SIRPB1, a variation was observed from aCGH and qPCR validation with 61% (28/46 OSCC samples) and 25% (12/48 OSCC samples) respectively.

### mRNA Expression of MGAM and ADAM9

**Figure 5:**
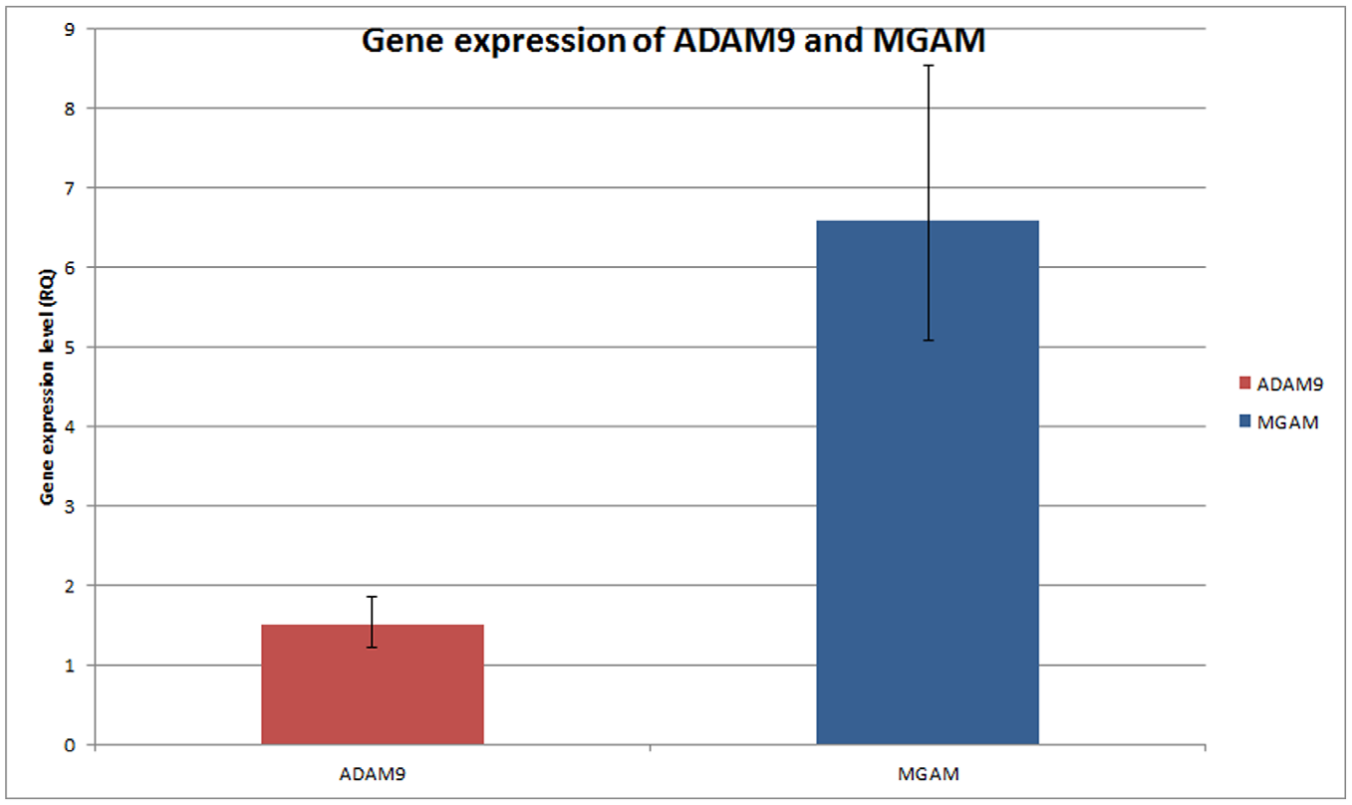
The gene expression level (RQ) of MGAM and ADAM9 in OSCC samples. The gene expression level (RQ) of MGAM and ADAM9 in OSCC samples based on the fold change which expressed as an average of 30 OSCC samples. In OSCC, MGAM has the highest gene expression level (RQ = 6.6) where the gene expression between OSCC and normal mucosa is statistically significant (p = 0.001). This is followed by ADAM9 with gene expression level of RQ = 1.51 (p = 0.093). The RQ for normal tissue (NT) of the two genes were 1 due to the normalization.

Both the MGAM and ADAM9 genes were singled out for further evaluation to confirm the genomic copy number changes and potential effects on gene expression. ADAM 9 was chosen due to its gene annotation, suggesting a high probability of involvement in tumorigenesis [20], [21]. Similarly, the MGAM gene has been reported to be amplified in gastric cancer [22]. In this study, qPCR analyses demonstrated that MGAM was over expressed in 29/30 OSCCs with a fold change of 6.6 (p = 0.001) whereas ADAM9 was over expressed in 23/30 OSCCs with a fold change of 1.51 (p = 0.093; Figure 5).

## Discussion

In this study, genome wide profiling was carried out using ultra-high-resolution aCGH to determine the genomic aberrations in a total of 46 OSCC samples. To the best of our knowledge, this is the first attempt to use ultra-dense aCGH technology for discovering CNAs within a large cohort of OSCC samples. As a result it is possible to identify CNAs down to a resolution of approximately 3 kb. This compares with the previous highest resolution study that used 105,000 probes [19]. Given the quantum level differential between this study and previous studies, concordance analysis in terms of CNA incidence, position and degree is challenging. However a broad meta-analysis of 38 previously published CNAs did present a good level of concordance at CNA level (25%), albeit with a high degree of heterogeneity in samples. This was particularly in relation to large CNAs such as CNAs as the following positions: 3p26.3-p11.2 (Deletion), 3q24-q29 (Amplification), 5p15.33-p11 (Amplification), 7p22.3-p11.1 (Amplification), 8p23.3-p11.1 (Deletion), 8q11.1-q24.4 (Amplification). These had been reported as arm level CNAs in previous studies, whereas we were able to identify intra-CNA focal, gains and losses, due to the enhanced resolution of the method used. Although overall for each previously published or “archetypal” CNA for OSCC, there was a representative finding in our study, we did observe samples that did not follow this model. A valid and probable explanation for this finding is that combining the enhanced resolution methodology with a large ethnically diverse cohort results in highly heterogeneous results. This has been also discovered for a wide variety of other cancers, where even within the same solid tumour, in the same individual, 5 different sub-types of cancer genomes were discovered [8].

Using this technology, few well documented CNAs were identified previously by others in OSCC that include amplification of 8q24 (MYC and PTK2), 3q24-29 (TERC, SOX2, EPHB3), 11q13 (PPFIA1, CTTN, FGF3, FGF4, FADD, CCND1), 11q22.3 (MMP7, MMP20, MMP27, MMP8, MMP10, MMP1, MMP3, MMP12, MMP13) and deletion of 3p26.3-p11.2 (FHIT, WNT5A) [19], [23], [24], [25], [26], [27], [28], [29]. Out of the common OSCC regions reported from Ambatipudi et al. [19] study, few common CNAs which showed high frequently amplification and deletion and their OSCC related genes were identified: 8q24.13-q24.3 (MYC, NDRG1, PTK2 and EXT1); 9q13-34.3 (CTSL1 and TNC); 11q23.3-q25 (ETS1); 14q21.3-q31.1 (HSP90AA1); 20q11.21-q13.33 (BCL2L1, TGIF2, TOP1) and 4q13.2 (UGT2B17). Through the basis of annotation suggesting that PTK2, CTTSL1, TNC, ETS1 and HSP90AA1 were involved in tumor cell proliferation, prevent tumor cell from apoptosis and promote tumor cell invasion in oral carcinogenesis [30], [31], [32], [33], [34], [35]. PTK2 encodes protein focal adhesion kinease which regulates cell adhesion in extracellular matrix and promote tumor invasion in oral carcinogenesis [31]. Activation of this gene also promotes cell proliferation and prevent tumor cell from apoptosis in OSCC [31]. Interestingly, Nagaraj and Zacharias [36] have showed that the stimulation of cigarette smoke would activate the proteolytic activity of CTSL1, which will degrade the matrix and enhance tumor cell invasion in oral carcinogenesis [33]. According to Jones and Jones [32] revealed that TNC is encoded as an ECM protein (tenascin-C) and the over-expression of this gene would enhance the tumor cell proliferation and invasion in tumorigenesis. Up-regulation of this gene has been found to correlate with tumor invasion in OSCC samples [33]. In Dittmer [34] study reported that over-expression of ETS1 would promote tumor invasion due to its ability to activate the MMP1, MMP3, MMP9 and uPA as well as of VEGF in tumorigenesis. HSP90AA1 encodes heat related proteins and activation of the chaperone protein would lead to cell proliferation and promote cell survival in tumorigenesis [35].

We also observed high frequency of amplification on chromosome 20q11.21-q13.33 in agreement with Sparano et al. [27] and Ambatipudi et al. [19] studies. Within this amplicon, BCL2L1, TGIF2, TOP1 genes were reported to be associated with OSCC. BCL2L1 plays an important role in apoptotic regulation that codes for anti-apoptotic and pro-apoptotic splice variant [37]. This gene was reported to be over-expressed in HPV-positive OSCC, when compared with HPV-negative OSCC using cDNA microarray [38]. Amplification of TGIF2 was reported to play a role in chromosomal instability in tumorigenesis indicating that this gene as a driver of chromosome 20q gain associated with oral carcinogenesis [39]. TOP1 gene was reported as amplified in colorectal cancer using fluorescent in situ hybridization (FISH) technique [40]. However, the mechanism of this gene remains unclear and further downstream analysis is recommended to clarify its role in oral carcinogenesis.

Using aCGH, we identified a complex pattern of amplifications and deletions of chromosome 4q13.2 in this study, which contained UGT2B17 gene. This phenomenon was also observed in Ambatipudi et al. [19] study, suggesting the tumor heterogeneity. UGT2B17 was reported as deleted in Jarvinen et al. [28] study which was mainly involved with tongue and larynx SCC. The complexity of differences in this gene reported in all these studies might be due to the employed OSCC samples different in term of socio-demographical and clinical such as risk habits (smoking, betel quid chewing and drinking alcohol) and tumor sites. The copy number polymorphism of this gene in cancer warrants further research into the role of this gene in OSCC.

In this study, we found amplification of 8p to be the most frequent event, being present in 80% (n = 37) of all OSCC included here. Genomic alterations at chromosome 8p have been frequently reported in human malignancy especially amplification of chromosome 8p11-12, which are rich with putative oncogenes [41], [42]. The entire gain of the p-arm of chromosome 8 has been implicated in many epithelial cancers, including breast cancer [43], [44]. This location is associated with several ADAMs family genes including ADAM9, ADAM5P, and ADAM32. ADAM family genes are generally transmembrane proteins, where its activation can lead to cell adhesion, proliferation, migration and proteolysis in cancer progression [19], [45]. The expression of ADAMs family gene has been identified with high-risk oral premalignant lesions, whereby it suggests the possibility of a role of these genes in OSCC transformation [46]. Another study by Sircoulomb et al. [47] has identified that amplification of 8p11.23 resulted in over-expression of the ERBB2 gene (an oncogenic tyrosine kinase) in estrogen receptor positive breast cancer.

Interestingly, a recent study using SNP array, on small cell lung cancer cell line, SCLC-21H, showed massively amplified segments of chromosome 8 that suggested this abnormality to be attributed to a mechanism termed chromothripsis, a phenomenon characterised by tens to hundreds of genomic rearrangements which take place in a ‘one off’ cellular crisis. It was suggested and showed by fluorescent in situ hybridization (FISH) karyotyping method that a possible mechanism for this, is that in the development of cancer, chromosome 8 is fragmented into pieces which are then stitched together into a derivative chromosome 8. The remaining fragments which are not stitched together would join to form a double minute chromosome. As this double minute chromosome contain genes such as MYC which would confer selective advantage to the daughter cells to multiply, additional internal rearrangements and over-replication, would result in the evolvement of massive amplification of chromosome 8 [48]. It is plausible that frequent amplification of 8 p as shown in this study might be due to this mechanism. Alternatively, as the entire p-arm is amplified, repeat elements at the centromere and p-telomere of chromosome 8 may be mediating its amplification by homologous recombination.

In this study cohort, high recurrent deletion of chromosome 3q26.1 was observed. Loss of 3q26.1 was observed in Familial Adenomatous Polyposis (FAP), an autosomal dominantly inherited form of colorectal cancer (CRC). In a study to identify a new cancer gene using genome-wide genotyping on mutation negative adenomatous polyposis coli (APC) gene in Familial adenomatous polyposis (FAP) family which was matched to on ethnicity and healthy controls, a CNA region at 3q26.1 was shown to be commonly lost in all polyps and was suggested to be precursors to CRC. This region was suggested to contain an element which is involved in the expression of an upstream tumor suppressor, PPM1L (protein phosphatase, Mg2+/Mn2+ dependent-like). PPM1L was quite recently discovered and characterised as a coding for a novel serine-threonine phosphatase in the oncogenic TGF-beta and BMP signalling pathways [49].

On chromosome 1q21.3-q22, a 3.5 Mb amplification contained more than 100 genes. These regions were reported to harbour potential oncogenes such as HAX-1, MUC1 and CKS1B genes, which have been previously reported as being amplified and over-expressed in OSCC samples [50], [51], [52]. HAX-1 is a HS1 associated protein X-1 which is reported to play an important role in protecting tumor cell from apoptosis and promote metastasis in breast cancer [53]. The over-expression of HAX-1 in OSCC is reported to promote tumor cell invasion by binding directly to beta6 and regulate the clathrin-mediated endocytosis of alphavbeta6 integrins in oral carcinogenesis [50]. MUC1 encodes a transmembrane glycoprotein that promotes the invasion of tumor cell and metastasis in tumorigenesis. Tsui and Garnis [54], reported, a complex pattern of amplification and deletion of this gene in tongue SCC cell lines using array CGH. Similarly, MUC1 was reported to be amplified and up regulated in advanced OSCC in an integrative study between copy number and gene expression of OSCC microarray data presented by Xu et al. [55]. The oncogenic function of MUC1 has been well reported in various cancers [56]. Recently, this gene has been suggested as an indicator for neck dissection for OSCC patients that were predicted to have lymph node metastasis [52]. CKS1B has been reported as an amplified gene in OSCC samples and over-expression of this gene could lead to the uncontrollable cell proliferation through dysregulation of the cyclin-dependent kinases (CDKs) in cell cycle progression in oral carcinogenesis [51].

We observed high amplification of chromosome 11q11 and 2p22.3 in the current study. Amplification of 11q11 contained olfactory receptors genes (OR4P1P, OR4S2, OR4C11, OR4C6 and OR4P4) and this region was reported to be amplified in OSCC samples [57]. Surprisingly, this region was reported amplified and deleted in different OSCC samples [19]. This suggested the possibility of copy number polymorphism of this region in OSCC genome. Similarly, amplification of chromosome 2p22.3 was identified in genome wide association study with oral cancer patients that were highly exposed to chewing tobacco [58]. This implies chewing tobacco could lead to CNA amplification in this region and lead to OSCC.

On chromosome 6p21.32, HLA-DRB5, HLA-DQB1 and HLA-DQA1 were identified as amplified in OSCC genome using aCGH. In Ambatipudi et al. [19] study, this region was reported amplified and deleted in among the OSCC samples differently by using the lower resolution oligonucleotide aCGH. However, Jarvinen et al. [28] reported that this region was deleted in the tongue SCC samples by using the lower resolution and sensitivity of BAC aCGH. This might implicate the existence of tumor heterogeneity and the possibility of contamination with normal and stromal cells in the tumor tissues that were tested in this study.

CNAs that identified from aCGH were further validated using qPCR method. In this study, qPCR copy number analysis on ADAM5P gene was carried out and more than 80% of the OSCC samples involved in aCGH (n = 46 OSCC) and qPCR (n = 48) showed amplified. Previously, ADAM5P have been reported as deleted in OSCC genome using the low resolution array CGH [19], [28]. The amplification of ADAM5P in more than 50% of the OSCC samples both in aCGH and qPCR further supported the evidence of tumor heterogeneity in OSCC. ADAM5P as the name implies is a pseudogene, which is located at chromosome 8p11.21. Only little is known about the possible role of pseudogenes in contributing tumorigenesis. However, study by Hirotsune et al. [59] has shown that pseudogene could regulate the expression of the gene by disrupting the transcription of the protein with an unclear mechanism.

The present study has identified a novel genomic amplification on chromosome 7q34 which was present in 34 out of 46 OSCC samples. Validation using qPCR showed that only 50% of the OSCC samples (n = 48) with MGAM copy number gain (2.25±0.18 copy numbers). This might be due to the dissimilarity between the array CGH platform and the qPCR based copy number assays as well as the differences is the intra-genic copy number variations of this gene [60]. There was no deletion identified for MGAM gene throughout the OSCC samples, using aCGH in our study. However, qPCR result showed more than 50% of the OSCC samples with low copy number gains (2.0–2.5 copy number). This could be due to the limitation of oligonucleotide platform of aCGH with its inability to identify the low-level copy number gains and deletions from the genome from the test samples [61]. The low copy number of gains detected from qPCR also might be due to the designation of qPCR assays that span the coding sequence of the candidate genes, while the aCGH probes span both coding and non-coding sequences [60].

In the present study, we observed a complex pattern of amplification (28/46) and deletion (14/46) of chromosome 20p13.1 using aCGH in correspondence to the SIRPB1 gene in different OSCC samples. The amplification and deletion of this gene have been reported in osteosarcoma, adenocarcinoma and chronic myeloid leukemia [62]. This can be explained by the copy number polymorphic characteristic of this gene [63]. These findings also showed the existence of tumor heterogeneity in tumor tissue. Validation using qPCR on this gene showed only 25% of the OSCC samples (n = 46) with amplification. The remaining samples were not determined, which suggested the homozygous deletion by this technique. Although the percentages to detect amplification of this gene in OSCC samples were small, it might suggest that the ultra-dense array CGH is more sensitive to detect the copy number alterations throughout the genome level. Recently, this technology has been introduced as a diagnostic tool for cancer and genetic disease. Another possible explanation might be due to the designation of qPCR assays that span the coding sequence of the SIBPB1 genes while the aCGH probes span both coding and non-coding sequences [60]. Ambatipudi et al. [19] has also revealed SIRPB1 in their genome wide profiling among Indian populations who were highly exposed to betel quid chewing. Genetic alteration of this gene has also been found in primary myelofibrosis [62] and colon cancer [64]. SIRPB1 is a member of the signal-regulatory protein (SIRP) family and the activation of this gene is involved in activating the SYK-JAK-STAT signalling, that regulates the proliferation and survival of cancer cells in tumor progression [62]. In view of this, the occurrence of this particular gene with oral cancers reflects the need for further studies on SIRPB1 genes to understand their role in oral cancer progression.

In this study, there is the lack of significant correlation association between the CNAs with socio-demographical and clinico-parameters which may be due to the limited power of the number of samples employed in this study. A larger set of OSCC samples are needed to yield significant CNAs that are associated with clinical outcomes. This is due to the fact that OSCC could be derived from different subsites within the oral cavity and form heterogeneous groups. Each of them behaved differently in clinical aspects and may have a different pattern of chromosomal aberrations.

It is well established that genomic amplification at gene(s) loci can increase gene dosage, which can lead to over-expression at mRNA level [65]. To characterise this, mRNA expression of ADAM9 and MGAM was quantified in 30 OSCCs. This comprised 19 samples involved in aCGH study and another independent set of 11 OSCC samples. These genes were chosen due to their high amplification frequency and also on the basis of their gene annotation, suggesting their likely involvement in tumorigenesis [21], [22]. ADAM9 has been previously implicated as a potential oncogene and therapeutic target for various cancers [21], [66] whilst MGAM gene has been reported to be amplified in gastric cancer genomes [22].

This study has identified a novel genomic amplification on chromosome 7q34 which was present in 34 out of 46 OSCC samples. The MGAM gene at this locus was significantly over expressed (6.6 fold) in 29 out of 30 samples analysed. It has been previously suggested that MGAM is a carbohydrate active enzyme that is involved in cell metabolism by breaking down the dietary starches and sugars into glucose [67]. The involvement of this gene in carcinogenesis could be explained by the Warburg effect which implies that during tumor progression, alterations are observed in glucose metabolism including glycolysis and oxidative phosphorylation process in cancer cells [68]. It could be hypothesized that over expression of MGAM may promote tumor growth by altering cell metabolism. Further investigation of this gene is required to elucidate its function, regulation and role in oral carcinogenesis.

In this study, ADAM9 gene was present on a genomic locus that was amplified on chromosome 8p11.23-11.22 whereas in the mRNA expression study, 76.67% (n = 23/30) OSCCs showed over expression of ADAM9 while the remaining OSCCs showed under-expression of this gene. Although the over-expression of ADAM9 was not at a significant level, the amplification of chromosome 8p was identified in 36/46 (80%) of OSCC samples. However, this finding was in contrast to the study conducted by Ambatipudi et al. [19] where they showed that ADAM9 was deleted in OSCC. This contradictory result could be due to the intra- and inter-tumor heterogeneity. Li et al. [69] hypothesized that these heterogeneities could give rise to different findings of gene expression and copy number alterations depending on the tumor sub-type. Interestingly despite the amplification, the gene itself did not appear to be up-regulated in all the OSCC samples when examined by RT-qPCR. This amplified gene could be silenced by other regulatory mechanisms such as epigenetics that could alter transcription into mRNA.

It is interesting to note the striking contrast between ADAM9 and MGAM. The former gene is located on a genomic amplification which spans the entire length of chromosome 8 p, hence likely to be driven by repeat elements at 8 p telomere and centromere, but does not have a significant impact on elevated expression of ADAM9 (less than 2 fold). MGAM on the other hand is located on a very precise, almost gene-specific genomic amplification on 7q34, and notably, is significantly over-expressed (more than 6 fold). Combining the finding of the precision, incidence (correlating to selective retention pressure of the CNA) and increase gene expression, MGAM could be a significant gene that drive OSCC development.

## Supporting Information

File S1 **Detailed list of candidate genes for the top most CNAs.**
(XLSX)
